# Quantitative proteomics of acutely-isolated mouse microglia identifies novel immune Alzheimer’s disease-related proteins

**DOI:** 10.1186/s13024-018-0266-4

**Published:** 2018-06-28

**Authors:** Srikant Rangaraju, Eric B. Dammer, Syed Ali Raza, Tianwen Gao, Hailian Xiao, Ranjita Betarbet, Duc M. Duong, James A. Webster, Chadwick M. Hales, James J. Lah, Allan I. Levey, Nicholas T. Seyfried

**Affiliations:** 1 Department of Neurology, Emory University, Atlanta, GA 30322 USA; 2 Department of Biochemistry, Emory University, Atlanta, GA 30322 USA

**Keywords:** Microglia, Neurodegeneration, Neuroinflammation, Immunology, Proteomics, Mass spectrometry, Alzheimer’s disease

## Abstract

**Background:**

Microglia are innate immune cells of the brain that perform phagocytic and inflammatory functions in disease conditions. Transcriptomic studies of acutely-isolated microglia have provided novel insights into their molecular and functional diversity in homeostatic and neurodegenerative disease states. State-of-the-art mass spectrometry methods can comprehensively characterize proteomic alterations in microglia in neurodegenerative disorders, potentially providing novel functionally relevant molecular insights that are not provided by transcriptomics. However, comprehensive proteomic profiling of adult primary microglia in neurodegenerative disease conditions has not been performed.

**Methods:**

We performed quantitative mass spectrometry based proteomic analyses of purified CD11b^+^ acutely-isolated microglia from adult (6 mo) mice in normal, acute neuroinflammatory (LPS-treatment) and chronic neurodegenerative states (5xFAD model of Alzheimer’s disease [AD]). Differential expression analyses were performed to characterize specific microglial proteomic changes in 5xFAD mice and identify overlap with LPS-induced pro-inflammatory changes. Our results were also contrasted with existing proteomic data from wild-type mouse microglia and from existing microglial transcriptomic data from wild-type and 5xFAD mice. Neuropathological validation studies of select proteins were performed in human AD and 5xFAD brains.

**Results:**

Of 4133 proteins identified, 187 microglial proteins were differentially expressed in the 5xFAD mouse model of AD pathology, including proteins with previously known (Apoe, Clu and Htra1) as well as previously unreported relevance to AD biology (Cotl1 and Hexb). Proteins upregulated in 5xFAD microglia shared significant overlap with pro-inflammatory changes observed in LPS-treated mice. Several proteins increased in human AD brain were also upregulated by 5xFAD microglia (Aβ peptide, Apoe, Htra1, Cotl1 and Clu). Cotl1 was identified as a novel microglia-specific marker with increased expression and strong association with AD neuropathology. Apoe protein was also detected within plaque-associated microglia in which Apoe and Aβ were highly co-localized, suggesting a role for Apoe in phagocytic clearance of Aβ.

**Conclusions:**

We report a comprehensive proteomic study of adult mouse microglia derived from acute neuroinflammation and AD models, representing a valuable resource to the neuroscience research community. We highlight shared and unique microglial proteomic changes in acute neuroinflammation aging and AD mouse models and identify novel roles for microglial proteins in human neurodegeneration.

**Electronic supplementary material:**

The online version of this article (10.1186/s13024-018-0266-4) contains supplementary material, which is available to authorized users.

## Background

Microglia are the primary innate immune cells of the central nervous system (CNS). These yolk sac-derived myeloid cells take up residence in the CNS during embryogenesis [1]. Microglia perform both homeostatic functions such as synaptic pruning and environmental sensing and under disease states, can adopt transcriptionally distinct profiles that can mediate both neurotoxic as well as neuroprotective and phagocytic roles [2–4]. In aging and in chronic neurodegenerative diseases such as Alzheimer’s disease (AD), microglia adopt unique disease-associated-microglial (DAM) transcriptional profiles and it is suspected that DAM can mediate phagocytic clearance of amyloid beta (Aβ), a process that becomes progressively dysregulated and ineffective with disease progression [5, 6]. Although transcriptional profiling provides an in-depth understanding of gene-expression changes in disease, modest agreement between transcriptional level changes and protein level changes have been observed across various studies and cell types [7, 8]. Since functional phenotypes are largely mediated by protein effectors, proteomic studies should provide an important perspective for understanding pathophysiological changes that result in neurodegeneration. Recent advances in mass spectrometry methods have made deep proteomic profiling of relatively smaller numbers of cells feasible [9].

The proteome of microglia from young and aging wild-type (WT) mice has been described [8, 10], and more recently, a limited proteomic analysis of acutely-isolated microglia from the 5xFAD mouse model of AD pathology identified early pro-inflammatory microglial activation prior to Aβ deposition [11]. However, comprehensive and comparative proteomic studies of adult microglia under homeostatic conditions, acute neuroinflammatory and chronic neurodegenerative disease states have not been reported. We report the deepest quantitative and most comprehensive proteome of acutely isolated microglia derived from adult mice and from mouse models of acute neuroinflammation and AD pathology. We report unexpected similarities between murine models of acute neuroinflammation and chronic neurodegeneration, in addition to highlighting unique neurodegeneration-specific immune changes seen in 5xFAD mice. We also highlight the complementary nature of transcriptomic and proteomic data in understanding the dynamic nature of microglial gene and protein expression changes under different in-vivo conditions. Furthermore, we identify unappreciated microglia-specific markers relevant to human AD as and suggest novel AD-relevant biology for proteins that were not thought to be expressed by microglia. Therefore, this microglial proteome represents a valuable resource to the neuroimmunology and neurodegenerative disease research communities, serving as a reference for future comprehensive immune profiling and target validation studies.

## Methods

### Animals

Female C57BL/6 J and female 5xFAD mice (on a C57BL/6 J background) used for the studies were housed in the Department of Animal Resources at Emory University under standard conditions. Institutional Animal Care and Use Committee approval was obtained prior to in-vivo work and all work was performed in strict accordance with the Guide for the Care and Use of Laboratory Animals of the National Institutes of Health. Adult mice were given intraperitoneal LPS injections (10 μg/dose × 4 daily doses) to induce acute neuroinflammation [12, 13]. If ≥25% of weight loss was observed, animals were euthanized. Three groups of adult 6–7 mo old C57BL/6 J mice (*n* = 3 pools each, 2 mouse brains per pool) were used for our studies. Wild-type (WT) mice (untreated), WT mice treated with LPS for 4 consecutive days (IP, 20 μg/dose) and 5xFAD mice were euthanized followed by cardiac perfusion as previously described [13].

### Acute isolation and enrichment of CD11b^+^ microglia and brain mononuclear phagocytes

The brain was dissected and brain mononuclear cells were isolated as previously described by Percoll gradient centrifugation [13]. Mechanical homogenization of the brain was performed rather than enzymatic digestion due to two reasons: i) in our experience, live microglial cell yield using enzymatic or mechanical dissociation methods are very similar (≈200,000 mononuclear cells per brain), and ii) enzymatic digestion may cleave cell surface proteins, thereby potentially decreasing the likelihood of identifying cell surface proteins by mass spectrometry. Isolated cells were then further purified by CD11b positive selection using a MACS column (Miltenyi Biotec Cat#130–093-636) to selectively enrich microglia and brain mononuclear phagocytes. Positive selection enriched the CD11b^+^ population from 60 to 95% as confirmed by flow cytometry. On average, 200,000 live CD11b^+^ cells were isolated from each brain. Whole cell microglial lysates were prepared and processed for analysis.

### Sample preparation for mass spectrometry analysis

Cell pellets were flash frozen and stored at − 80 °C until ready for protein extraction. Cells were lysed in 8 M urea lysis buffer (8 M urea, 100 mM NaHPO4, pH 8.5) with HALT protease and phosphatase inhibitor cocktail (ThermoFisher). Each sample was then sonicated for 3 cycles consisting of 5 s of active sonication at 30% amplitude followed by 15 s on ice. Samples were then centrifuged for 5 min at 15,000 g and the supernatant was transferred to a new tube. Protein concentration was determined by bicinchoninic acid (BCA) assay (Pierce) and 20 μg of each sample was aliquoted.

Protein digestion and sample cleanup was performed as previously published [14]. Briefly, protein samples were reduced with 1 mM dithiothreitol (DTT) for 30 mins, alkylated with 5 mM iodoacetamide (IAA) in the dark for another 30 mins and then 8-fold diluted with 50 mM triethylammonium bicarbonate (TEAB). Overnight digestion was performed with 1:100 (*w*/w) Lysyl endopeptidase (Wako) followed by an additional 12-h digestion with Trypsin at 1:50 (w/w). The peptide solutions were acidified, and desalted with a C_18_ Sep-Pak column (Waters). A 2 μg equivalent of each sample elution was pooled and used to create a global internal standard (GIS) and all samples were dried under vacuum.

Tandem mass tag (TMT) peptide labeling was performed according to manufacturer’s instructions and as previously described [14]. One batch of 10-plex TMT kits (Thermo Fisher) was used to label the 9 samples and one GIS mixtures. In this batch, TMT channel 131 was used to label the GIS standard, while the 9 remaining TMT channels were used to label each individual sample. Electrostatic repulsion-hydrophilic interaction chromatography (ERLIC) offline fractionation was performed as previously described [14, 15]. Briefly, dried samples were re-suspended in 100 μL of ERLIC buffer A (90% acetonitrile with 0.1% acetic acid) and separated on a PolyWAX LP column (20 cm by 3.2 mm packed with 300 Å 5 μm beads (PolyLC Inc.) and elution fractions were recovered over a 45-min gradient from 0 to 50% ERLIC buffer B (30% ACN with 0.1% FA). The original 44 fractions were combined as previously described into 21 and dried under vacuum [14, 15].

### Mass spectrometry analysis and TMT data acquisition

Assuming equal distribution of peptide concentration across all ERLIC fractions, 10 μL of loading buffer (0.1% TFA) was added to each of the fractions and 2 μL was separated on a 25 cm long by 75 μm internal diameter fused silica column (New Objective, Woburn, MA) packed in-house with 1.9 μm Reprosil-Pur C_18_-AQ resin. The LC-MS/MS platform consisted of a Dionex RSLCnano UPLC coupled to an Orbitrap Fusion mass spectrometer with a Flex nano-electrospray ion source (Thermo Fisher). Sample elution was performed over a gradient of 3 to 30% Buffer B (0.1% formic acid in ACN) over 105 mins (flow rate started at 300 nl/min and ended at 350 nl/min), from 30 to 60% B over 20 mins at 350 nl/min, and from 60 to 99% B over 5 mins at 350 nl/min. The column was equilibrated with 1% B for 10 min at a flow rate that increased from 350 nl/min to 400 nl/min. The MS was operated in positive ion mode and utilized the synchronous precursor selection (SPS)-MS3 method for reporter ion quantitation as described [14]. The full scan range was 380–1500 m/z at a nominal resolution of 120,000 at 200 m/z and automatic gain control (AGC) set to 2 × 10^5^. Collision-induced dissociation (CID)-Tandem MS/MS at 35% normalized collision energy (CE) and higher energy collision dissociation (HCD) SPS-MS3 at 65% normalized collision energy (CE) were collected at top speed with 3 s cycles. For SPS, the top 10 product ions were notched and fragmented.

### Protein identification and quantification

Raw data files from Orbitrap Fusion were processed using Proteome Discover (version 2.1). Collected MS/MS spectra were searched against the UniProt mouse proteome database (54,489 total sequences). SEQUEST parameters were specified as: trypsin enzyme, two missed cleavages allowed, minimum peptide length of 6, TMT tags on lysine residues and peptide N-termini (+ 229.162932 Da) and carbamidomethylation of cysteine residues (+ 57.02146 Da) as fixed modifications, oxidation of methionine residues (+ 15.99492 Da) and deamidation of asparagine and glutamine (+ 0.984 Da) as a variable modification, precursor mass tolerance of 20 ppm, and a fragment mass tolerance of 0.6 Da. Peptide spectral match (PSM) error rates were determined using the target-decoy strategy coupled to Percolator [16] modeling of true and false matches. Reporter ions were quantified from MS3 scans using an integration tolerance of 20 ppm with the most confident centroid setting. An MS2 spectral assignment false discovery rate (FDR) of less than 1% was achieved by applying the target-decoy strategy. Following spectral assignment, peptides were assembled into proteins and were further filtered based on the combined probabilities of their constituent peptides to a final FDR of 1%. In cases of redundancy, shared peptides were assigned to the protein sequence with the most matching peptides, thus adhering to principles of parsimony. The search results and TMT quantification as well as raw LC-MS/MS files are included in the ProteomeXchange online repository with identifier PXD009137. Even though TMT labeling limits missing values, normalized abundances were zero for some channels in the dataset, up to 3 out of the 9 channels representing individual samples. Missing values were imputed according to the informative missing-ness assumption, such that these values fit into the left tail of the population-wide Gaussian distribution, with a mean 1.8 population-wide standard deviations less than the mean for non-missing data, and randomness of the imputed population was allowed to vary within ±0.3-fold of the same standard deviation.

### Gene ontology enrichment analysis and visualization, pathway analysis and identification of upstream transcriptional regulators

Functional enrichment of the differentially expressed proteins was determined using the GO-Elite (v1.2.5) python package [17]. The set of total proteins identified and quantified (*n* = 4133) was used as the background. Input lists included proteins significantly differentially expressed (*p* < 0.05 by Student’s t test) comparing either 5xFAD vs WT or LPS-treated WT vs untreated WT mouse microglial proteomic data. Z-score determines over-representation of ontologies and Fisher’s exact test *p*-value was used to assess the significance of the Z-score. GO Elite v1.2.5 command line application with Z score cutoff of 1.96 and minimum 5 genes per ontology identified ontology enrichments for LPS or AD model-affected proteins, similar to our previously published method [18]. Enrichment Map v2.1.0 Cytoscape plugin [19] was called from Cytoscape v3.5.1 on DAVID-formatted GO-Elite pruned Z-Score output tables. Edge connectivity (Jaccard similarity metric) ranges from 0 (white) to 1 (dark red or blue). Ontology network node colors represent the degree of average log_2_ fold change for all differentially expressed members of the ontology in the AD-WT and LPS-WT/CT networks. Since no overlap of gene symbols occurs between differential expression list-derived up and down subnetworks, respectively red and blue subnetworks were overlaid to produce the visualizations shown. Differentially expressed protein lists were also used for bioinformatics analysis (MetaCore software, Thomson Reuters) including pathway analyses to identify over-represented molecular and metabolic pathways as well to identify potential upstream transcriptional regulators as previously described [20].

### Immunofluorescence microscopy

For mouse tissue, brains were harvested after cardiac perfusion with ice cold saline, followed by 4% paraformaldehyde. After overnight fixation, brains were transferred to 30% sucrose for 24 h after which 30 μm sections were obtained using a cryostat. For all immunohistochemistry studies, three sagittal fixed brain sections from wild-type and 5xFAD mice from equivalent regions were placed on each slide to control for any heterogeneity in staining. For Aβ retrieval, sections were treated with 70% formic acid for 10 min, washed in buffer, blocked with 3% hydrogen peroxide and 10 μg/ml of Avidin for 30 min and then blocked in 10% normal horse serum prepared in Tris-buffered saline (TBS) for 30 min followed by overnight incubation with primary antibody (anti-Aβ42 4G8 antibody [Signet, Cat # 9220–02] 1:1000, anti-Apoe antibody [Meridian Life Science, Cat # K74180B] 1:100 [21], anti-Iba1 antibody [Abcam ab178846], anti-GFAP [Millipore Sigma, Cat # MAB360] 1:100, anti-CD68 [Abcam ab955] 1:100). Sections were rinsed in TBS and then incubated in the appropriate fluorophore-conjugated secondary antibody (1:500) for 30 min after which tissues were mounted on slides, dried and mounted with mounting medium containing DAPI for nuclear staining (Fluoreshield, Sigma-Aldrich F6057). Confocal microscopy was performed on Leica SP8 multi photon microscope and all image processing was performed using Imaris software. For co-localization analyses, appropriate thresholds for each channel were determined using appropriate negative controls. Immunofluorescence microscopy was performed on an immunofluorescence microscope (Microscope: Olympus BX51 and camera: Olympus DP70) and image processing was performed using ImageJ.

### Immunohistochemistry (IHC)

Cryopreserved pathology-confirmed AD (*n* = 3) and age- and sex-matched non-disease control (n = 3) frontal cortex tissues were obtained from the Emory Neuropathological core and sectioned using a cryotostat (30 μm slice thickness). Endogenous peroxidase was quenched with 0.3% hydrogen peroxide for 30 min and then the sample was blocked with 10% normal goat serum/0.4% Triton X-100/Tris-buffered saline (TBS) for 1 h, followed by avidin/biotin binding (Vector labs SP2001) per manufacturer’s instructions, followed by overnight incubation with primary antibodies (anti-Cotl1 [Sigma-Aldrich HPA008918, also validated in the Human Protein Atlas] rabbit polyclonal Ab 1:100) [22]. Sections were rinsed with TBS and incubated with appropriate biotin-conjugated secondary antibody followed by Vectastain Elite ABC and diaminobenzidine (DAB) as per manufacturer’s recommendations. To exclude nonspecific staining unrelated to polyclonal and monoclonal antibodies, immunostaining was performed with omission of the antibodies but with all other procedures unchanged. Slides were lastly counterstained with hematoxylin. Light microscopy was performed with an Olympus light microscope (Olympus, Center Valley, PA). Images acquired from 4 AD cases and 4 non-AD/non-disease post-mortem cases (frontal cortex) were used for quantitative analysis to compare Cotl1 immunoreactivity (ImageJ software) as described [23].

### Existing proteomic and transcriptomic datasets used for comparative analyses

#### Primary microglial proteomics data

An existing proteome of acutely isolated and purified microglia as well as other brain cell types (neurons, astrocytes and oligodendroglia) [8] was used as a reference proteome. Although mice used for our study were significantly older (6–7 mo) compared to this previously published work (8 weeks), we used this existing proteome as a reference to identify microglial-specific proteins as well as the most abundant proteins identified in microglia. We also referenced this dataset to serve as a background of all identifiable proteins in mouse brain.

#### Purified microglial transcriptomics

CD11b^+^ microglia microarray datasets from wild-type and 5xFAD mice were obtained from GEO dataset GSE65067 [24]. Existing RNAseq datasets comparing wild-type to LPS-treated mice were also obtained [25]. Differentially expressed genes were contrasted with observed differentially expressed proteins comparing wild-type to LPS-treated wild-type microglia as well as comparing wild-type vs. 5xFAD microglia.

#### Human post-mortem brain proteomics data

Protein expression data were obtained from ProteomeXchange (http://proteomecentral.proteomexchange.org/cgi/GetDataset?ID=PXD007160) [14]. In this study, TMT mass spectrometry was used to identify 10,230 proteins across AD, Parkinson’s disease and non-disease control post-mortem brain (frontal cortex) tissues. Protein expression data from AD (*n* = 10) and non-disease controls (*n* = 10) were used for differential expression analyses, which were then used for comparison with the current mouse microglial proteome.

### Other statistical considerations

Graphpad Prism (Ver. 5), Microsoft Excel and SPSS (Ver. 22) were used to create graphs and perform statistical analyses. All data are shown as mean ± SEM. Pairwise two-tailed t-tests were performed in unadjusted analyses comparing groups. Statistical significance was set at *p* value ≤0.05 for all experiments unless specified separately.

## Results

### Quantitative proteomics of acutely isolated adult mouse microglia reveals a highly metabolically active phenotype

**Fig. 1:**
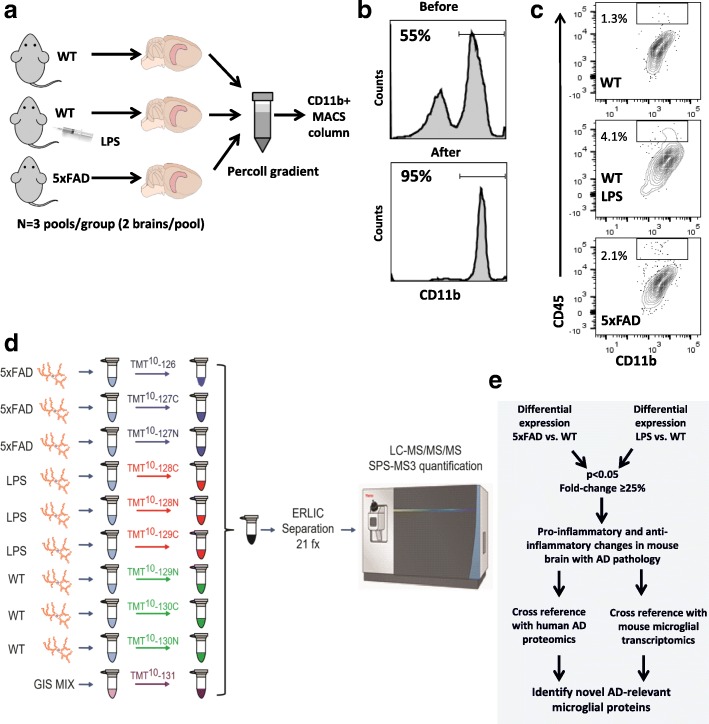
Study design and analytic approach for comprehensive quantitative proteomic analysis of murine microglia. **a** Work-flow summarizing isolation and purification of acutely isolated CD11b^+^ CNS immune cells from untreated 6–7 mo WT (C57BL/6 J) mice, LPS-treated WT mice (4 daily i.p. doses, 20 μg/dose) and age/sex-matched 5xFAD transgenic mice were (*N* = 3 pools per group, 2 brains per pool). Following percoll density centrifugation, cells were enriched for CD11b^+^ cells by MACS magnetic separation. **b** Flow cytometric confirmation of successful enrichment of CD11b^+^ cells after MACS enrichment. Pre-enrichment (top), CD11b^+^ cells account for 50–60% of all live CNS mononuclear cells. Post-enrichment (bottom), CD11b^+^ cells account for > 95% of all live cells. **c** Flow cytometric confirmation of minimal presence of peripherally derived CD11b + CD45high CNS-infiltrating macrophages or perivascular macrophages in untreated WT, LPS-treated WT and 5xFAD mice. **d** Proteomic work-flow for tandem mass tag (TMT) mass spectrometry based quantification. **e** Analytic approach used for differential expression analyses of datasets

To describe the proteome of adult mouse microglia, we analyzed whole cell lysates of acutely isolated CD11b^+^ MACS-purified microglia isolated from brains of 6–7 mo old wild-type (WT) mice treated with saline or intra-peritoneal LPS for 4 days to induce neuroinflammation [13], as well as from 5xFAD mice (Fig. 1a, 2 brains pooled per sample, *n* = 3 samples per group) [26]. MACS purification resulted in > 95% enrichment of CD11b^+^ CNS immune cells (Fig. 1b). Among CD11b-enriched cells, CD45^high^ mononuclear phagocytes accounted for only 1.3, 2.1 and 4.1% cells in untreated WT, LPS-treated WT and 5xFAD mice respectively (Fig. 1c) confirming minimal contamination by CNS-infiltrating macrophages. Microglial whole-cell lysates in 8 M urea buffer were digested by LysC and trypsin, followed by labeling of peptides with TMT reagents, admixture of the TMT 10-plex peptides, followed identification and synchronous precursor selection based MS3 (SPS-MS3) mass spectrometry reporter quantitation (Fig. 1d), and then differential expression and comparative analyses (Fig. 1e).

**Fig. 2:**
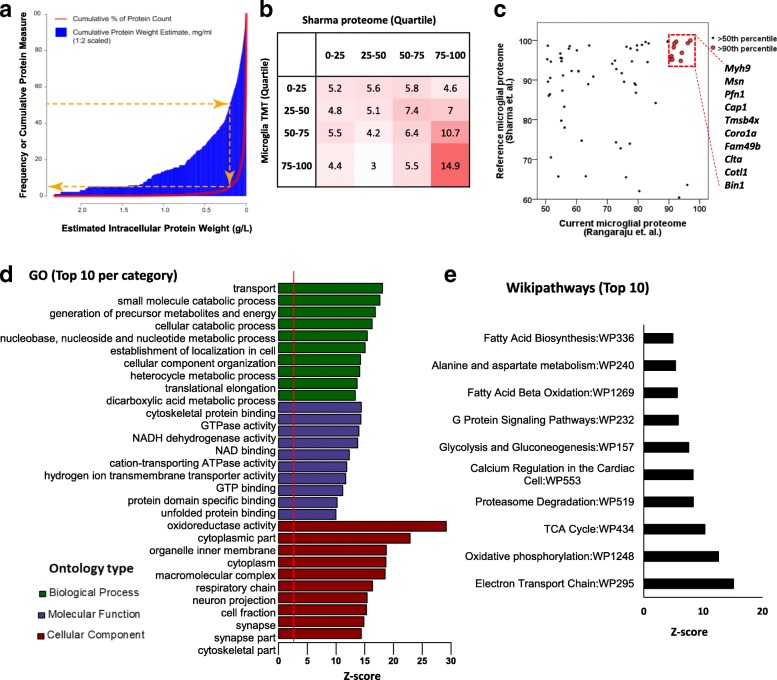
A high-level summary, gene ontology and pathway analysis of 4133 microglial proteins identified by TMT. **a** Histogram representing the distribution of microglial proteins sorted by protein wet weight (bins with proteins contributing the most wet weight are on the left). Individual protein wet weight contributions were estimated using the Perseus Proteomic Ruler analysis plugin [66], which uses measurements of histone abundance to normalize reporter abundances assuming a total wet weight density of 200 mg/ml. The cumulative distribution of protein abundance was highly dependent on a minority of proteins (of 4133 total) such that a majority of cumulative protein mass was accounted for by a small number of proteins, i.e., 5.1% of all proteins accounted for 50% of cumulative protein mass. **b** Comparison of relative abundance (abundance percentile rank) of proteins identified in our dataset with a reference mouse microglial proteome [8]. Abundance values of proteins identified in WT mouse microglia were binned into quartiles and compared with the reference proteome. The proportions (%) of microglial proteins are shown in each box. **c** Scatter plot representation of the most abundant proteins identified in our microglial proteome and the reference microglial proteome. Proteins that were among the top 10th percentile of abundance in both datasets are highlighted in red. **d** Results from Gene Ontology (GO) enrichment analysis of 4133 identified microglial proteins against a background list of all known mouse gene symbols (or 11,937 proteins in a total brain reference proteome) [8]. The top 10 GO terms from each of the three GO groups (Green: biological process, Purple: molecular function, Brown: cellular component) are shown. Degree of enrichment of each GO term is indicated by the Z-score (X-axis). Nearly identical results were obtained either background list of all mouse gene symbols or 11,937 mouse proteins identified in a total brain reference proteome. **e** Top 10 enriched functional pathways (WikiPathways) identified by enrichment analysis of 4133 microglial proteins

We obtained a total of 31,865 unique peptides that mapped to 4259 unique mouse gene symbols. Of these, 4133 proteins were identified across the three groups (see Additional File 1: Table S1).. Not allowing any missing values, 3598 proteins were quantified. The distribution of protein abundance was highly skewed such that 5% of proteins (*n* = 211) accounted for 50% of the cumulative protein weight in the samples (Fig. 2a). Most of these highly abundant proteins were housekeeping and cytoskeletal proteins such as Atp1a3 (α3 subunit of Na^+^/K^+^ ATPase), Sptan1 (α spectrin), Actb (β actin), Sptbn1 (β spectrin) and Tubb4b (beta4 tubulin). Using a previously published purified mouse brain and cell type-specific proteome as a reference (7,198 identified and quantified proteins) [8], 3379 proteins identified in our dataset were also identified in the reference microglial proteome. Of these, 156 proteins were highly abundant (≥90th percentile of abundance) in both datasets. Overall, there was modest correlation of expression levels between the reference microglial proteome and our microglial proteome (Fig. 2b, Spearman’s rank correlation *R* = 0.28 for all proteins in common, Spearman’s *R* = 0.23 for the top 25th percentile in common). This modest correlation suggests possibility of methodological and age-related differences in microglial protein expression and myelination, since our proteome was derived from significantly older mice (26–30 weeks of age) as compared to the reference proteome (8–9 weeks of age). In the reference proteome, 633 microglia-specific proteins were also identified as having highest expression in microglia as compared to neurons or other glial cells. Of these, 189 proteins were identified in our microglial dataset. Among the most highly abundant proteins in both microglial proteomes (≥80th percentile of abundance), 17 proteins also met criteria for microglia-specific expression (Fig. 2c). These proteins consistent across two independent datasets (including Myh9, Msn, Coro1a, Cotl1 and Bin1), represent the most highly abundant and microglial-specific proteins in mice.

We also performed gene ontology (GO) enrichment analyses on the entire list of 4133 proteins and identified 180 significant GO terms (adjusted *p*-value < 0.05). The top 10 GO terms for each category are shown in Fig. 2d (also see Additional file 2: Table S2). This GO analysis shows that our microglial proteome was enriched for cytosolic/cytoplasmic proteins involved in transport and several catabolic, nucleotide/nucleoside metabolic and mitochondrial processes. Interestingly, “neuron projection” and “synapse” were also identified as significantly enriched GO terms in this dataset, which may be explained by the known role of microglia in neuronal synaptic pruning [27, 28]. WikiPathways enriched in our microglial proteome included electron transport chain, oxidative phosphorylation/TCA cycle, proteasome degradation, glycolysis/gluconeogenesis, fatty acid beta oxidation and G protein signaling pathways (Fig. 2e, Additional file 2: Table S2). Results from GO analyses were not affected by the choice of background proteins used (all known mouse gene symbols or 11,937 proteins identified in a reference mouse brain proteome) [8]. In summary, the global microglial proteome from adult mice shows that the large majority of microglial proteins are ubiquitously expressed housekeeping proteins although highly abundant and microglia-specific proteins also exist. The observed enrichment of glycolytic, proteasome degradation, mitochondrial oxidative phosphorylation and fatty acid beta oxidation pathways suggest that adult microglia represent a highly metabolically active and dynamic group of glial cells, rather than bystander resting cells as they were once perceived to be.

### Differential expression and comparative analyses of microglia in mouse models of acute neuroinflammation, AD pathology and advanced aging

**Fig. 3:**
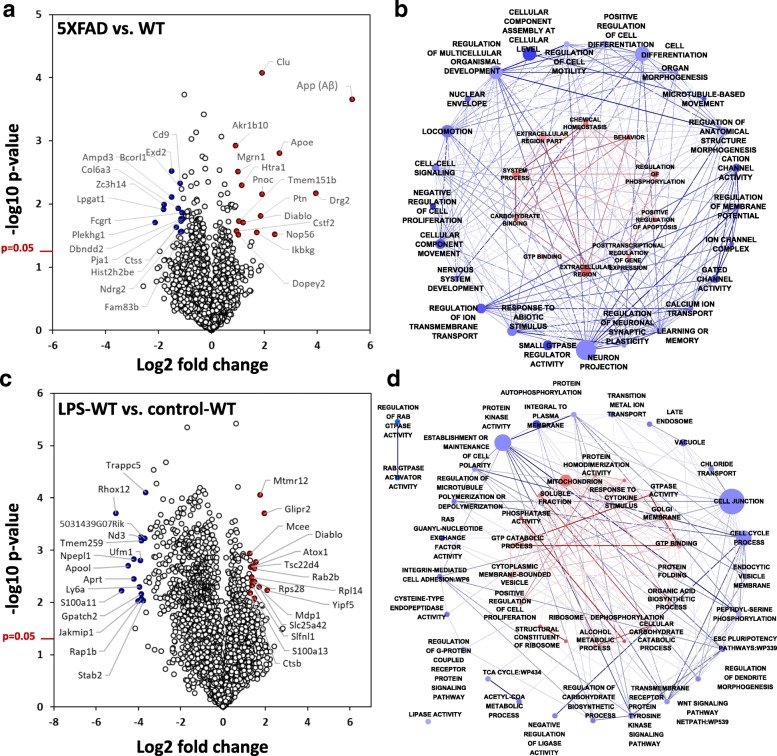
Differential expression analyses of proteins identified in microglia derived from homeostatic, LPS-induced neuroinflammation and 5xFAD neurodegeneration mouse models. **a** Volcano plot representation of differentially expressed proteins comparing 5xFAD with untreated WT mouse microglia. The top 15 proteins with increased (red) and decreased (blue) expression are shown. X-axis represents log_2_ transformed fold change (5xFAD vs WT) and y-axis represents -log_10_ transformed *p*-value from a pairwise two-tailed T test comparing the two groups. **b** Gene enrichment map summarizing Gene Ontology (GO) terms and WikiPathways enriched in the 5xFAD vs WT differential expression analysis. Enrichment Maps were created in Cytoscape (Enrichment map plugin) Cytoscape using DAVID-formatted GO-Elite pruned Z-Score output tables. Red or Blue colors indicate significant GO terms (Z-score > 1.96) enriched in differentially expressed protein lists in 5xFAD microglia vs. WT microglia (Red: increased, Blue: decreased). Edge connectivity (Jaccard similarity metric) ranges from 0 (light color intensity) to 1 (dark red or blue intensity). Ontology network node colors represent the degree of average log2 fold change for all differentially expressed members of the ontology in the AD-WT networks. Since no overlap of gene symbols occurs between differential expression list-derived up and down sub-networks, respectively red and blue sub-networks were overlaid. Scale AD-CT: bluest (− 0.75) < −> 0 (white) < −> reddest (2.27 log_2_Fold change). **c** Volcano plot representation of differentially expressed proteins comparing LPS-treated WT with untreated WT mouse microglia. The top 15 proteins with increased (red) and decreased (blue) expression are shown. X-axis represents log_2_ transformed fold change (LPS-treated WT vs untreated WT) and y-axis represents -log_10_ transformed p-value from a pairwise two-tailed T test comparing the two groups. **d** Gene enrichment map summarizing GO analysis of differentially expressed proteins in LPS-treated WT mouse microglia. Gene enrichments were created as described above using significant GO terms enriched in differentially expressed protein lists comparing microglia isolated from LPS-treated WT and untreated WT mice (Red: increased in LPS-treated WT mice; Blue: decreased in LPS-treated WT mice) microglial proteins. Scale LPS-WT/CT: bluest (− 2.21 log2FC) < −> 0 (white) < −> reddest (1.22 log_2_ fold change)

Of the 4133 proteins identified across all three groups, 187 proteins were differentially expressed in 5xFAD microglia and 1230 proteins were differentially expressed following LPS treatment in WT mice (Fig. 3, Additional file 3: Figure S1). Of 187 proteins differentially expressed in 5xFAD mice (Fig. 3a), 56 proteins demonstrated ≥1.25-fold increased expression (top 5: App, Drg2, Apoe, Nop56 and Clu) while 105 proteins demonstrated ≥1.25-fold decreased expression (top 5: Plekhg1, Lpgat1, Zc3h14, Ampd3 and Exd2). By applying Benjamini-Hochberg false-discovery-rate (FDR) correction to group-wise ANOVA *p*-values, 71 proteins demonstrated significant differential expression (FDR < 0.05) across the three groups. Of these, 23 proteins were differentially expressed comparing 5xFAD to WT microglia (6 up-regulated and 17 down-regulated) and the 6 proteins with increased expression in 5xFAD microglia included App, Apoe, Clu, Htra1, Rab2b and Necab1. GO analysis of differentially expressed proteins showed that extracellular proteins, carbohydrate binding, post-transcriptional regulation, GTP binding and regulation of phosphorylation were specifically enriched in proteins increased in 5xFAD microglia while extracellular proteins, anatomic structure development, ion channel activity, transmembrane transport inhibition of cell proliferation, locomotion and learning and memory were enriched GO terms in proteins decreased in 5xFAD microglia (Fig. 3b, Additional file 4: Table S3). Pathway analyses of differentially expressed proteins identified aberrant lipid trafficking, altered lipid metabolism and ATP metabolism as being enriched among proteins with increased expression in 5xFAD microglia while anti-inflammatory IL4 signaling was enriched in proteins with decreased expression in 5xFAD microglia (Additional file 5: Table S4). Transcriptional factors predicted to be upstream regulators of proteins overexpressed in 5xFAD microglia included PUR2α, AP-2 and p53 while Dec1 (Stra13) and c-Myc were predicted as upstream regulators of proteins decreased in 5xFAD microglia (Additional file 4: Table S3).

**Fig. 4:**
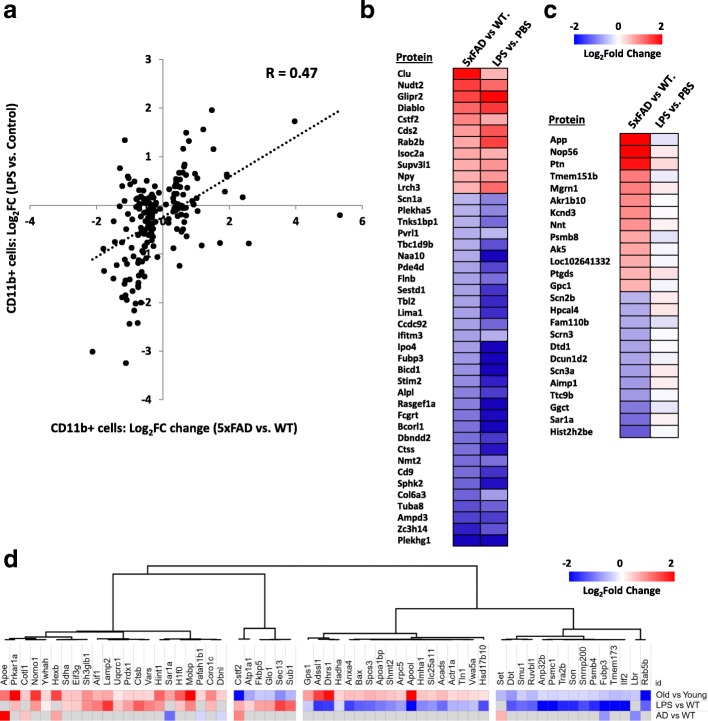
Comparison of proteomic profiles of LPS-induced pro-inflammatory and 5xFAD AD pathological microglial states. **a** Scatter plot representation of relative expression (log2 transformed fold change) of all proteins differentially expressed in both LPS-treated WT (vs. untreated WT) as well as 5xFAD (vs WT) mouse models. Overall Pearson’s correlation coefficient was 0.47, *p* < 0.001. **b** Heat map representing proteins with concordant changes in expression in microglia isolated from LPS-induced pro-inflammatory and 5xFAD neurodegeneration mouse models (compared to untreated WT microglia). **c** Heat map representing protein expression changes seen only in 5xFAD microglia but not in LPS-treated WT microglia. **d** Comparison of proteomic profiles of microglia from advanced aged mice with microglia derived from acute neuroinflammatory and 5xFAD neurodegeneration mouse models. Ageing-associated proteins and their relative expression data were obtained from an existing microglial proteome from young (age 3–4 mo) and aged (age 20–24 mo) WT C57BL/6 J mice [10]. Aging-associated proteins that were differentially expressed in either LPS-treated WT (vs WT) or 5xFAD (vs WT) conditions were included and an unsupervised hierarchical clustering analysis was performed to identify 4 distinct clusters. Grey boxes indicate proteins that were not significantly altered in the respective comparison

In addition to identifying neurodegeneration-associated microglial proteomic changes, we used the systemic LPS-induced neuroinflammation model to compare acute neuroinflammatory changes with chronic neuroinflammation seen in more indolent neurodegeneration models [12, 13]. In microglia from LPS-treated WT mice, 363 proteins were ≥ 1.25-fold upregulated and 823 were ≥ 1.25-fold down-regulated (Fig. 3c). Proteins increased following LPS treatment were enriched for GO terms including cellular responses to stress/cytokines, mitochondrial localization, increased cell proliferation and ribosomal processes (Fig. 3c, Additional file 4: Table S3). Conversely, proteins decreased by LPS treatment were enriched for GO terms including oxidoreductase activity, cell adhesion and cell contact, carboxylic acid metabolism and ATP metabolism, suggesting a general suppression of oxidative phosphorylation and motility (Fig. 3d, Additional file 2: Table S2). We identified several pro-inflammatory LPS-upregulated microglial proteins that were increased in 5xFAD microglia (top 5: Clu, Nudt2, Glipr2, Diablo and Cstf2), and 30 LPS-downregulated proteins that were decreased in 5xFAD microglia (top 5: Bcorl1, Plekhg1, Rasgef1a, Ipo4 and Bicd1). Proteins differentially expressed in 5xFAD mouse microglia showed mostly concordant directions of change in microglia derived from LPS-treated WT mice (Fig. 4a, *R* = 0.47 *p* < 0.001) suggesting that AD pathology, in general, induces a pro-inflammatory state in microglia. However, differences between LPS-activated microglia and 5xFAD microglia were also observed. Several proteins were differentially expressed in only in 5xFAD mouse microglia without any LPS-induced changes, including increased levels of App (Aβ peptide), Nop56, Ptn, Tmem151b and Mgrn1 and decreased levels of Hist2h2be, Sar1a, Ttc9a and Aimp3 (Fig. 4c), suggesting that these proteins may be uniquely relevant to AD pathology. A comparison of GO enrichment terms from each analysis (5xFAD vs WT and LPS-WT vs WT, Fig. 3) reveals that while both LPS and AD pathology resulted in a general inhibition of microglial motility/locomotion, LPS-treatment induced proliferative responses and mitochondrial metabolic processes while AD pathology inhibited cell differentiation and proliferation while promoting apoptosis, highlighting key metabolic and functional differences between these two states of microglial activation despite anatomical/structural similarities.

To compare the proteomic profile of microglia from acute neuroinflammation and neurodegeneration models with that of advanced aging, we contrasted our findings with an existing microglia proteomic study that compared young (3–5 mo) and very old mice (20–24 mo) [10]. Of 267 aging-associated microglial proteins identified in this reference dataset, 198 were identified in our microglial proteome of which 62 were differentially expressed either in 5xFAD or LPS-treated WT mouse microglia. These 62 proteins could be broadly classified into 4 clusters based on direction of change in protein expression in aged and LPS-treated mice (Fig. 4d). Only 8 proteins with altered expression in 5xFAD microglia also showed aging-related changes. Of the proteins increased in 5xFAD microglia, Hexb, Apoe and Cotl1 increased with aging while Cstf2 and Set decreased with aging. Interestingly, all three proteins that increased in aged WT and 5xFAD mouse microglia are highly expressed by microglia in the CNS [29], highlighting the microglial specificity of these AD- and aging-related protein alterations. In summary, this comparative microglial proteomic analysis from models of acute neuroinflammation, aging and AD pathology demonstrate model-specific as well as overlapping inflammatory changes in microglia.

### Assessment of concordance between transcriptomic and proteomic changes in microglia in acute neuroinflammatory and neurodegenerative disease states

**Fig. 5:**
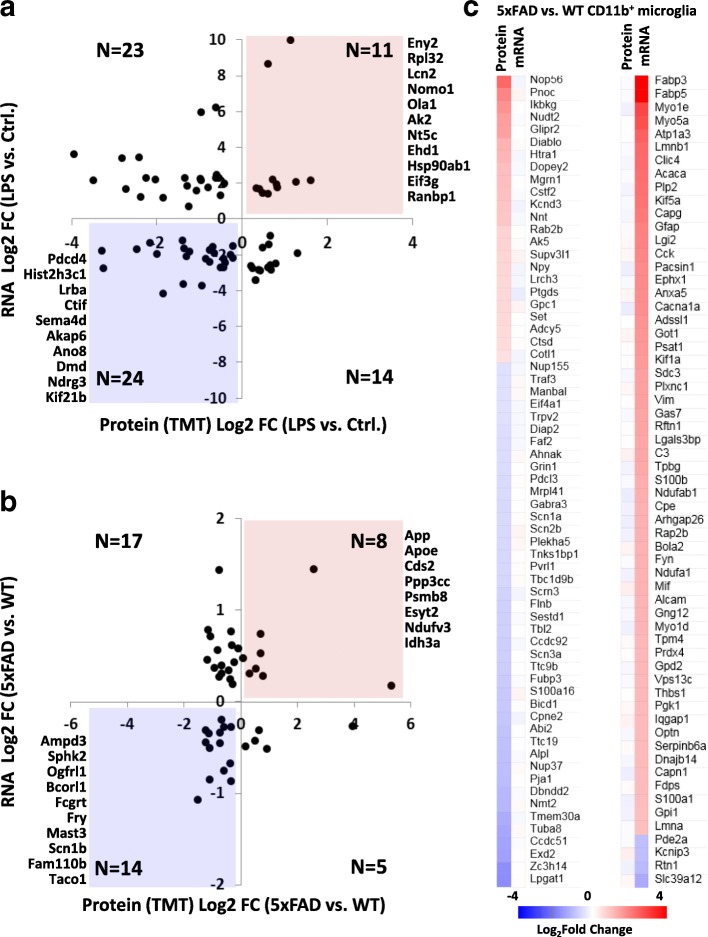
Modest concordance between transcriptomic and proteomic changes in microglia in acute neuroinflammatory and neurodegenerative disease conditions. **a** Scatter plot showing level of concordance between differentially expressed proteins and differentially expressed gene transcripts comparing LPS-treated WT to untreated WT microglia. The current microglial dataset was compared to transcriptomic data for shared gene symbols in a previously published RNA sequencing study of acutely isolated microglia from WT and LPS-treated WT mice [25]. Only differentially expressed proteins/transcripts in both datasets are shown. Gene symbols in the top right or bottom left quadrants indicate concordant changes. **b** Scatter plot showing level of concordance between differentially expressed proteins and differentially expressed gene transcripts comparing 5xFAD to WT microglia [24]. Our microglial proteomic dataset was cross referenced with an existing microarray gene expression study of acutely isolated WT and 5xFAD mouse microglia. Concordant changes are indicated in the top right and bottom left quadrants. **c** Heat map representing expression changes in 5xFAD microglia (vs. WT microglia) seen only at the protein level (left) or at the transcriptomic level (right)

To assess the degree of concordance between transcriptomic and proteomic changes in microglia, we contrasted our proteomic findings in WT, LPS-induced neuroinflammation and 5xFAD models with existing RNAseq or microarray data from CD11b^+^ microglia acutely isolated from similar model systems [5, 25]. Of the common gene symbols in existing RNAseq and our proteomic dataset from LPS-treated WT mouse microglia, we identified 72 genes/proteins were differentially expressed in both datasets of which only 35 (48.6%) showed concordant changes (11 up-regulated, 24 down-regulated, Fig. 5a). Similarly, we contrasted our proteomics findings to existing transcriptomic data obtained from CD11b^+^ microglia from WT and 5xFAD mice [24], in which 3200 genes were found to be differentially expressed. Our proteomic dataset comparing 5xFAD to WT microglia included 902 of the 3200 genes which were altered at the transcript level. Among the 187 differentially expressed proteins (Fig. 5b), 43 were also altered at the transcriptomic level. Of these 43, only 22 (51.2%) showed concordant changes (8 up-regulated, 14 down-regulated). Among gene symbols identified in both proteomics and transcriptomics datasets, we also identified expression changes in 5xFAD mice that occurred exclusively at the protein or transcript levels (Fig. 5c). Proteins upregulated in 5xFAD microglia that did not demonstrate changes at the transcript level included Nop56, Pnoc, Ikbkg, Nudt2, Glipr2, Diablo and Htra1. Conversely, genes upregulated in 5xFAD microglia without proteome-level changes included Fabp3, Fabp5, Myo1e, Myo5a, Atp1a3, Lmnb1 and Clic4. Overall, these results highlight the modest levels of concordance between expression changes at the transcriptome and proteome levels and emphasize the need to perform molecular profiling at both transcript and protein levels since each can provide unique and novel biological insights into neurodegenerative disease processes.

### Identification of microglial proteomic changes in AD mouse models that are relevant to human AD

**Fig. 6:**
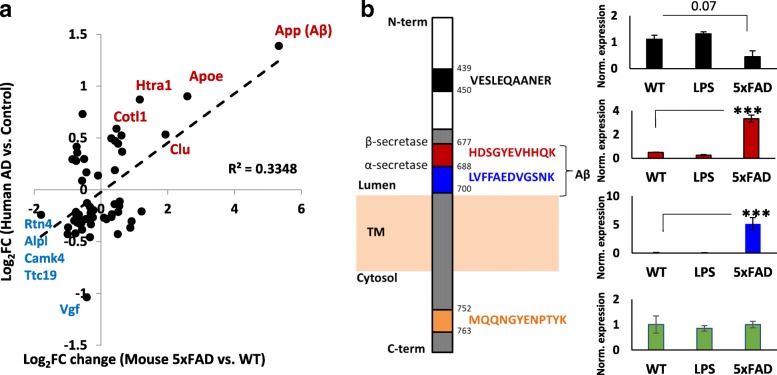
Identification of microglial proteins with increased expression in 5xFAD mouse microglia with relevance to human AD pathology. **a** Scatter plot showing relative expression of proteins that were differentially expressed in both 5xFAD (vs WT) mouse microglia as well as a published human AD (vs non-AD non-disease control) whole brain proteomics dataset [18]. Pearson’s correlation coefficient was determined to assess overall concordance. The top 5 concordantly increased (red) and decreased (blue) proteins are highlighted. Proteins shown in red indicate increased expression while blue proteins indicate decreased expression in both datasets. **b** Peptide-level analysis of all App peptides identified in the microglial proteome. Each unique peptide sequence is shown with a distinct color and each peptide’s expression across all 3 mouse groups (relative to global internal standard) is shown. * *p* < 0.05, **p < 0.01, ***p < 0.005

Since amyloid β accumulation seen in 5xFAD mice only represents one of the pathological hallmarks of human AD, we do not expect all protein changes in mouse 5xFAD microglia to be relevant to human disease [26]. To identify proteomic changes observed in mouse 5xFAD microglia that are consistent with human AD, we compared our mouse microglial proteome with an existing human post-mortem brain proteome in which frontal cortex from non-disease controls and pathology-confirmed AD cases were compared [14]. Three thousand six hundred forty-four proteins identified in our microglial proteome were also identified in this reference human brain proteome. While the overall correlation between relative protein expression data between mouse microglia and human brain samples for all identified proteins was low (R^2^ = 0.002), moderate correlation (R^2^ = 0.34) was observed for 55 proteins that were differentially expressed in both datasets. Of these 55 proteins, 11 (including App, Apoe, Htra1, Cotl1 and Clu) were concordantly increased while 23 (including Vgf, Rtn4, Alpl, Scn3a and Camk4) were concordantly decreased in both 5xFAD microglia and human AD brain (Fig. 6a).

Since App was identified as the most highly differentially expressed and human AD-relevant protein in 5xFAD microglia, we further investigated individual App peptide-level expression to determine whether this finding was driven by de-novo App synthesis or Aβ phagocytosis by microglia. Five unique App peptides were identified in the dataset (Fig. 6b) of which 2 mapped to the Aβ sequence, 2 mapped to the C-terminal YNPTY endocytosis motif and 1 mapped to N-term residues 439–450. Since all five App peptides were identical in mice and humans, we were unable to directly confirm the source (mouse or human) of the App peptides identified in microglia. Selective increased expression of both Aβ42 peptides was noted in 5xFAD microglia while the C-term peptide and the N-term sequences showed no group-wise differences. This pattern of App peptide differential expression strongly suggests that intra-microglial Aβ is phagocytosed and not produced within microglia.

### Validation of microglial Apoe and Cotl1 protein expression in AD

**Fig. 7:**
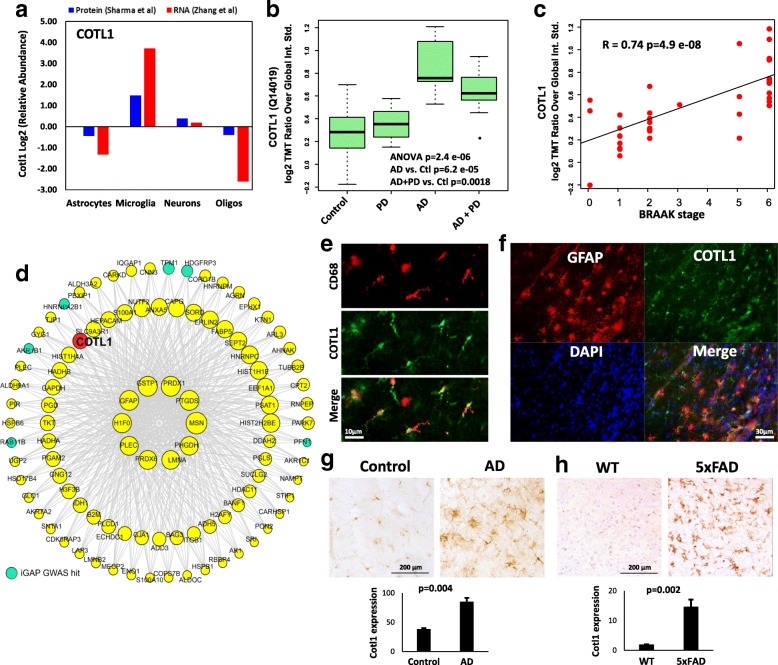
Validation of Cotl1 as a novel microglia-specific protein with pathophysiological significance in human AD. **a** Comparison of Cotl1 transcript and protein expression in CNS cells (astrocytes, microglia, neurons and oligodendrocytes). Data were derived from existing brain cell-type specific RNA sequencing and proteomics datasets [8, 29]. **b** Cotl1 protein expression in post-mortem human brain (frontal cortex) comparing non-disease controls, pathologically confirmed AD, clinically confirmed cases of Parkinson’s disease (PD) and patients with both AD and PD (*n* = 10 individuals per group). ANOVA and post-hoc pairwise t-test *p*-values are shown. Protein-level expression data from human post-mortem brains was obtained from a published proteomic dataset [18]. **c** Correlation between Cotl1 protein expression in post-mortem human brain with neurofibrillary tangle neuro-pathological grade (BRAAK stage). Correlation coefficient (R) and significance of the association are shown. **d** Cotl1 was identified as a member of a glial-enriched protein module identified by weighted co-expression network analysis of human post-mortem brain proteomic data from 40 cases (10 controls, 10 AD, 10 PD and 10 AD+PD) [18]. Human AD risk genes identified by MAGMA analysis of AD genome wide association studies (iGAP study) in this module are indicated (light blue). **e** Immufluorescence micrographs from post-mortem human brain (frontal cortex) showing microglial marker CD68 (Red) and Cotl1 expression (Green). Co-localization between CD68 and Cotl1 is shown in the merged channel below. **f** Immunofluorescence micrographs from post-mortem human brain (Frontal cortex) showing astrocytic Gfap (Red) and Cotl1 (Green) expression (nuclei in Blue: DAPI). Lack of co-localization between Gfap and Cotl1 is shown in the merged channel. **g** Immunohistochemistry micrographs from AD and non-disease control post-mortem human brains (frontal cortex) showing increased microglial Cotl1 expression (Brown) in AD as compared to non-disease controls (representative images from *n* = 4 AD and n = 4 non-AD cases). Quantitative analysis of Cotl1 immunoreactivity is shown (right)

Among the top 5 proteins (Fig. 6a) with increased expression in 5xFAD microglia and post-mortem human AD brain, Apoe and Cotl1 were also highly expressed at the transcriptional level by microglia [8, 29]. Therefore, we performed additional neuropathological studies to characterize Cotl1 and Apoe protein expression in AD. Cotl1 or coactosin like F-actin binding protein 1 is an actin-binding protein that promotes the activity of 5-lipoxygenase, as enzyme that converts arachidonic acid into leukotriene A4 [30]. Cotl1 is also expressed by immune cells including macrophages and has been recently reported to regulate actin dynamics at the immune synapse in addition to promoting the function of lipoxygenase 5 (5LOX), an enzyme that converts arachidonic acid to leukotriene A4 (LTA4) [31]. Cotl1 is highly and specifically expressed by microglia at the transcript and protein levels (Fig. 7a) [8, 29], consistent with our finding that Cotl1 is a highly abundant microglial protein. In an analysis of an existing proteomic dataset comparing frontal cortical regions from AD, Parkinson’s disease and non-disease post-mortem brain tissues [18], we found that Cotl1 protein expression was increased in AD cases (Fig. 7b). Cotl1 protein expression was also strongly correlated with neurofibrillary tangle pathology (Braak stage) (Fig. 7c) and belonged to a glial co-expression protein module that was positively associated with AD pathology (Fig. 7d) [32, 33]. In addition to Cotl1, this glial module also contained other proteins that are pertinent to eicosanoid biosynthesis such as LTA4 hydrolase (Lta4H) which catalyzes the conversion of LTA4 to LTB4 [34] and, prostaglandin D2 synthase (Ptgds) which catalyzes the conversion of prostaglandin H2 (PG-H2) to PG-D2 [35]. In immuno-histochemical studies of human post-mortem brains, we found that Cotl1 was specifically expressed by CD68^+^ microglia (Fig. 7e) but not by GFAP^+^ astrocytes (Fig. 7f). In a comparative immunohistochemistry analysis of AD and non-AD post-mortem frontal cortex tissues, we found that cortical and sub-cortical white matter microglia showed significantly higher Cotl1 protein expression as compared to non-AD controls (Fig. 7g). We also observed that microglia highly expressing Cotl1 in the AD brain had an activated morphology with larger cell bodies and numerous processes (Fig. 7g).

**Fig. 8:**
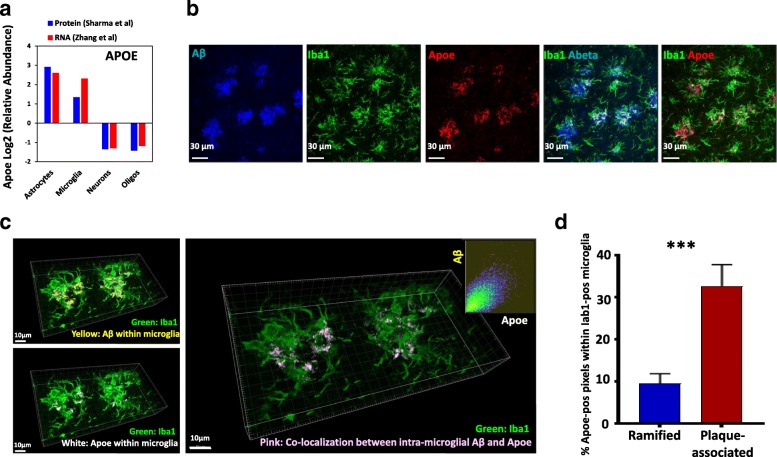
Apoe protein is present within and compartmentalized with Aβ within plaque-associated microglia in 5xFAD mouse brain. **a** Comparison of Apoe transcript and protein expression in CNS cells (astrocytes, microglia, neurons and oligodendrocytes). Data were derived from existing brain cell-type specific RNA sequencing and proteomics datasets [8, 29]. **b** Immunofluorescence micrographs (maximal projections of Z-stack confocal microscopic images) showing Aβ (4G8, Blue), Iba1 (Green) and Apoe (Red) expression in 5xFAD mouse brain. Overlap between Iba1 and Aβ (White) and between Iba1 and Apoe (White) are shown in two-channel merged images. **c** 3D reconstructions of 0.5 μm thick confocal microscopic Z-stack images from 5xFAD mouse brains showing intra-microglial Aβ (Yellow) and intra-microglial Apoe (White). Co-localization between intra-microglial Aβ and Apoe is shown in light pink (right). Degree of co-localization (correlation) between Apoe-positive and Aβ-positive pixels is shown in the inset image (R^2^ = 0.88). **d** Comparison of intra-microglial Apoe in ramified microglia (wild-type mice) with Aβ plaque-associated microglia (5xFAD mice). Proportion of Apoe+ pixels within Iba1+ microglia (ramified or plaque-associated) was determined by co-localization analysis as shown in panels (**b**) and (**c**). N=5 images (subiculum of the hippocampal formation) from 3 WT and 3 5xFAD mice were obtained (*** p < 0.005)

The second protein we validated was Apoe (apolipoprotein E) which regulates cholesterol and Aβ transport into the brain and is strongly implicated in AD pathogenesis [36]. Carriers of the Apoε4 allele have significantly higher risk of developing late-onset AD and in mouse models of AD pathology, Apoε4 promotes neuroinflammatory responses leading to neurodegeneration, independent of its effects of Aβ processing [6, 36, 37]. Apoe is highly expressed in astrocytes and microglia at both transcriptomic and proteomic levels (Fig. 8a) and is a specific marker of disease-associated-microglia that are highly prevalent in neurodegenerative diseases [5, 8, 29]. We observed that Apoe protein expression in mouse 5xFAD brains closely resembled immunostaining of Aβ plaques (Fig. 8b) which was not affected by preincubation with Aβ fibrils (Additional file 3: Figure S2). Apoe also partly co-localized with Iba1 immuno-reactive microglia (Fig. 8b) confirming the presence of intra-microglial Apoe protein. Although Apoe and Aβ (4G8) showed overlapping plaque-like immunostaining patterns with high degree of co-localization, Apoe within microglia also co-localized with Aβ, suggesting shared intracellular compartmentalization, presumably within phagosomes (Fig. 8c and d). These data suggest that Apoe within microglia could represent either phagocytic co-uptake of Apoe along with Aβ, or may represent functional interaction between microglial Apoe in the phagocytic uptake of extra-cellular Aβ and shared subsequent subcellular compartmentalization in microglia. These results show that Cotl1 is specific microglial protein that is selectively increased in AD while Apoe, at least at the protein level, may be involved in phagocytic uptake of Aβ in plaque-associated microglia.

## Discussion

Advances in deep RNA sequencing and proteomic methods have provided unprecedented opportunities to characterize the molecular and cellular complexity of the CNS [5, 8, 9, 25, 32, 38, 39]. Microglia are the principal immune effectors in the CNS [1] that perform homeostatic functions under normal conditions but adopt diverse activated profiles as revealed by recent whole-cell and single-cell transcriptomic studies [5, 6, 8, 25]. Several transcriptomic changes in microglia, however, may not reflect changes at the protein level due to post-transcriptional events that further regulate protein expression and degradation, leading to modest levels of agreement between transcript and protein expression data [7, 8, 32]. Primary microglial proteomes from 8-week-old wild-type mice [8] and aged mice [10] as well as young adult 5xFAD mice (2–12 weeks of age) have been published [11]. However, proteomic profiling of microglia from neuroinflammation models and later time points in neurodegenerative disease models has not been reported, representing a significant gap in the field. We present a comprehensive proteome of acutely isolated CD11b^+^ microglia from older adult mice (6–7 mo og age) under normal, acute neuroinflammatory and chronic neurodegenerative disease conditions and highlight differentially expressed proteins specific to each state with relevance to human AD.

By contrasting microglia-specific changes in 5xFAD mice to existing human brain proteomes, we have found that App, Apoe, Clu, Htra1 and Cotl1 were among the most highly upregulated proteins in microglia isolated from a mouse model of AD pathology. Of these, App, Apoe, Clu and Htra1 have known AD-relevant biology although their expression and role within microglia in AD is not clear [36, 40, 41]. Increased expression of Apoe, Htra1 and Clu proteins, but not of Cotl1, has been reported in 5xFAD microglia as early as 10 weeks of age when Aβ deposition is first seen [11]. Additionally, we identified Cotl1 as a microglia-specific marker with increased expression in human AD. Cotl1 was also found in a recent network analysis study of post-mortem human AD brains to be a key member of a glial module that is positively correlated with AD pathology [32]. In this module, Cotl1 was co-expressed with leukotriene A4 hydrolase (Lt4ah). Since Cotl1 stabilizes and promotes the activity of lipoxygenase 5 (5-LOX) to convert arachidonic acid to LT-A4 [30, 31], a co-upregulation of Lt4ah in AD microglia is predicted to facilitate LT-A4 conversion to LT-B4, a pro-inflammatory leukotriene implicated in neurotoxicity and Aβ formation [42–44]. Another protein co-expressed with Cotl1 was PG-D2 synthase (Ptgds), an enzyme that catalyzes the conversion of PG-H2 to PG-D2 in the cyclooxygenase pathway of arachidonic acid metabolism [45]. Non-specific upstream inhibition of cyclooxygenase-2 (COX2) as well as specific inhibition of PG-D2 signaling results in neuroprotection in animal models [46, 47]. These converging lines of evidence suggest that at least some pro-inflammatory effects of microglia in AD may be mediated by LTB4 and PG-D2 [45]. Since Cotl1 is involved in actin dynamics, immune synapse function and regulation of eicosanoid biosynthesis, we suspect that Cotl1 may regulate motility and neuroinflammatory functions mediated by microglia in AD [30, 31, 45].

Apoe expression, like Cotl1, was also highly increased in 5xFAD microglia. This is consistent with observations from transcriptomic studies of microglia where Apoe was upregulated in neurodegenerative disease models and was identified as a marker of disease-associated-microglia [5, 6, 20]. Our immuno-histochemical studies show that Apoe protein, unlike Cotl1, is not expressed by ramified/homeostatic microglia, but is seen within microglial processes that surround and infiltrate Aβ plaques in 5xFAD mice, and specifically co-localizes with intra-microglial Aβ. Since activated microglia engulf Aβ and Apoe strongly co-localizes with Aβ within microglia, our findings suggest that microglia may either phagocytose Aβ and Apoe that co-aggregate in the AD brain or that Apoe’s co-localization with Aβ indicates functional interaction between Aβ and Apoe during Aβ phagocytosis and clearance [48, 49]. In support of the latter possibility are recent findings demonstrating the role of Apoe, along with Trem2, in the transition of microglia from homeostatic to DAM states in AD [27, 29], as well as the consistent identification of Apoe within microglia in our as well as other microglia proteomes [8, 10, 11].

In addition to validating specific proteins with increased expression in 5xFAD microglia, our bioinformatics analyses provide clues regarding the general metabolic and inflammatory states of microglia in AD. We observed that dysfunctional lipid metabolism and trafficking seem to be features of microglia in AD brain. Since dysfunction in lipid metabolism and clearance are implicated in AD pathogenesis [50, 51], comprehensive lipidomic characterization of microglia from AD models may provide novel insights into mechanisms of impaired lipid clearance in AD. Our observation of general suppression of IL4-related pathways in 5xFAD microglia along with similarities between protein expression profiles of microglia in 5xFAD mice and LPS-induced neuroinflammatory states suggest that microglia in AD adopt a profile more consistent with that of pro-inflammatory microglia. However, the identification of p53 and AP2 as upstream transcriptional regulators of AD-associated microglial activation are distinct from regulators of canonical LPS-induced pro-inflammatory activation such [52–54] suggesting that unique inflammatory activation pathways are also upregulated by microglia in AD models. Beyond changes in AD models, our results from LPS-treated WT mice also demonstrate robust effects of systemic inflammation on energy metabolism and oxidative stress in microglia. GO analyses showed that LPS-induced systemic inflammation suppressed mitochondrial oxidative phosphorylation in microglia. In LPS-treated mouse microglia, 21 mitochondrial proteins were reduced while cellular stress response proteins were upregulated, suggesting that stress-induced mitochondrial damage may occur in microglia in response to systemic inflammation. Furthermore, LPS upregulated proteins involved in focal adhesion formation, proliferation, exocytosis and apoptosis confirming a state of microglial activation [13]. Since systemic LPS does not cross the blood brain barrier [55], our observations are more likely to represent indirect effects of LPS (involving Tnf or other soluble factors) rather than direct toll-like-receptor-mediated effects of LPS on microglia [56].

Our study also identified several highly abundant and microglia-specific proteins including Bin1, Msn, Cotl1 and Hexb [3]. The identification of Bin1 as a highly abundant protein in our microglial proteome agrees with a reference mouse microglial proteome [8] and is of particular relevance in AD [57]. At the transcript level, Bin1 is highly and specifically expressed by homeostatic microglia and Bin1 down-regulation occurs as microglia adopt DAM profiles in neurodegenerative disease [5, 25]. Although Bin1 polymorphisms have been identified as independent risk factors for late-onset AD [57], the pathophysiological roles of Bin1 in AD are unclear. Based on our findings that are consistent with existing expression data for Bin1 in the brain [8, 25], it may be particularly important to further investigate the role of Bin1 in microglia-mediated neuroinflammatory mechanisms in neurodegenerative disorders. We also identified Hexosaminidase B (HexB) as abundant microglial protein that was also previously shown to be microglia-specific at transcriptomic and proteomic levels [3, 5, 8, 25]. We observed that HexB was upregulated by 5xFAD microglia. Hexb is an endosomal-lsysomal enzyme that regulates ganglioside metabolism and is involved in autophagy [58]. Hexb mutation results in a rare lysosomal storage disorder called Type II GM2 gangliosidosis or Sandhoff disease that is characterized by progressive hearing loss, seizures, intellectual disabilities and motor deficits [59]. In the Hexb knock-out mouse, accumulation intra-neuronal Aβ-like and synuclein-like protein aggregates [58]. Our findings of Hexb upregulation in microglia from AD mouse models further support the possibility that dysregulation of lipid metabolism, lysosomal functions and autophagy in microglia may contribute to AD neuropathology [60–62].

Despite the identification of microglia-specific proteins discussed above, some of the more well-known microglial proteins such as Tmem119 and P2ry12 were not identified in our study [63, 64]. It is also interesting to note that the reference microglial proteome [8] did not identify Tmem119 in microglia even though Tmem119 protein expression by microglial has been confirmed by immuno-histochemical methods [25, 64]. This may be explained by our use of whole cell lysates, rather than membrane-enriched fractions for our study, leading to a lower likelihood of identifying cell membrane anchored proteins. This bias in our dataset is further supported by the lack of enrichment of GO terms such as “cell membrane” in our analyses. Another possibility for the relative absence of membrane proteins in our study is potential loss of cell membrane fragments during the cell isolation process, a likely result of heavy myelination and architectural complexity of the aging mouse brain as contrasted with post-natal or younger adult mice. Since membrane receptors, ion channels and transporters may represent drug targets for neurodegeneration, future studies using more advanced proteomics technologies with deeper coverage coupled with enrichment of membrane-associated microglial proteins will likely help overcome this limitation.

Additional limitations of this study also deserve discussion. In addition to ubiquitous and microglia-specific proteins, several proteins highly expressed by astrocytes, oligodendrocytes and neurons were also identified in our microglial proteome despite > 95% enrichment of CD11b^+^ cells in our samples. While this could indicate cell or protein contamination by adherent non-microglial cell fragments during CD11b^+^ enrichment, this could also be indicative of constant pruning of neuronal synapses or dendrites as well as phagocytic uptake of cellular debris, myelin and astrocytic processes by microglia. Indeed, an age dependent increase in myelin basic protein (Mbp) peptides in mouse microglia has been previously reported [65], which is more likely to explain why Mbp was highly abundant in our dataset (top 10 percentile). As compared to the reference proteome in which over 7000 proteins were identified across 4 cell types by label-free quantitative methods [8], we identified 4133 proteins by TMT. This lower number of proteins identified could be attributed to lower microglial yield from the older mouse brain as compared to younger mice. Thus, pooling additional brain samples as well as enzymatic digestion prior to microglial isolation may be considered in future studies to improve protein yield. A more likely explanation for lower number of proteins identified is that our single batch of fractionated TMT, 10-plex-quantified by SPS-MS3 mass spectrometry did not contain non-microglial enriched samples from which to borrow or trigger identification and quantification of shared proteins which may be higher expressed in other cell types (i.e., neurons, oligodendrocytes and astrocytes). Lastly, our analyses using 3 pools per group were statistically underpowered to detect statistically significant changes using FDR corrections for pair-wise comparisons because of which we considered unadjusted *p*-values < 0.05 as statistically significant. To overcome this inherent limitation, we applied an additional fold-change threshold of 25% which exceeded the overall coefficient of variation for all protein measurements (6–7%) in each of the three groups.

## Conclusions

In conclusion, this deep and comprehensive proteomic study of purified adult mouse microglia has allowed us to identify shared and unique microglial proteomic changes in acute neuroinflammatory, aging and AD mouse models in addition to identifying novel roles for microglial proteins in human neurodegeneration. In addition to serving as a valuable resource to the neuroscience research community, our study emphasizes the value of applying state-of-the-art proteomics methods as a complimentary approach to transcriptomics, to provide novel molecular insights in neurodegeneration.

## Additional files

Additional file 1: **Table S1.** Log2 transformed protein abundance data and differential expression analyses. (XLSX 1522 kb)

Additional file 2: **Table S2.** GO Elite analysis of all 4,133 proteins identified in mouse microglia by TMT mass spectrometry. (XLSX 63 kb)

Additional file 3: **Supplemental Figures.** **Figure S1.** Hierarchical clustering of proteins differentially expressed (*p*<0.05) in either WT-LPS vs WT-control and 5xFAD vs WT comparisons. **Figure S2.** Pre-incubation of anti-Apoe antibody with fibrillar Aß does not abolish plaque-like immunostaining for Apoe. (DOCX 761 kb)

Additional file 4: **Table S3.** GO Elite analysis of differentially expressed proteins in WT, LPS-treated WT and 5xFAD mouse microglia. (XLSX 20 kb)

Additional file 5: **Table S4.** Pathway analysis of proteins differentially increased in 5xFAD (vs. WT) microglia. (XLSX 18 kb)

## Abbreviations

AD: Alzheimer’s disease
Aβ: Amyloid beta
DAM: Disease-associated-microglia
LPS: Lipopolysaccharide
