# Supplementary material for: Quantitative proteomics of acutely-isolated mouse microglia identifies novel immune Alzheimer’s disease-related proteins
Source: Mol Neurodegener. 2018 Jun 28;13:34. doi: 10.1186/s13024-018-0266-4 (PMC6025801; doi:10.1186/s13024-018-0266-4)
Supplement: Supplementary file 3 — Supplemental Figures. Figure S1. Hierarchical clustering of proteins differentially expressed (p<0.05) in either WT-LPS vs WT-control and 5xFAD vs WT comparisons. Figure S2. Pre-incubation of anti-Apoe antibody with fibrillar Aß does not abolish plaque-like immunostaining for Apoe. (DOCX 761 kb) [file 13024_2018_266_MOESM3_ESM.docx]

**Quantitative proteomics of acutely-isolated mouse microglia identifies novel immune Alzheimer’s disease-related proteins**

Authors

Srikant Rangaraju, Eric B Dammer, Syed Ali Raza, Tianwen Gao, Hailian Xiao, Ranjita Betarbet, Duc Duong, James A Webster, Chadwick M Hales, James J Lah, Allan I Levey, Nicholas T Seyfried

**Supplemental Figures 2**


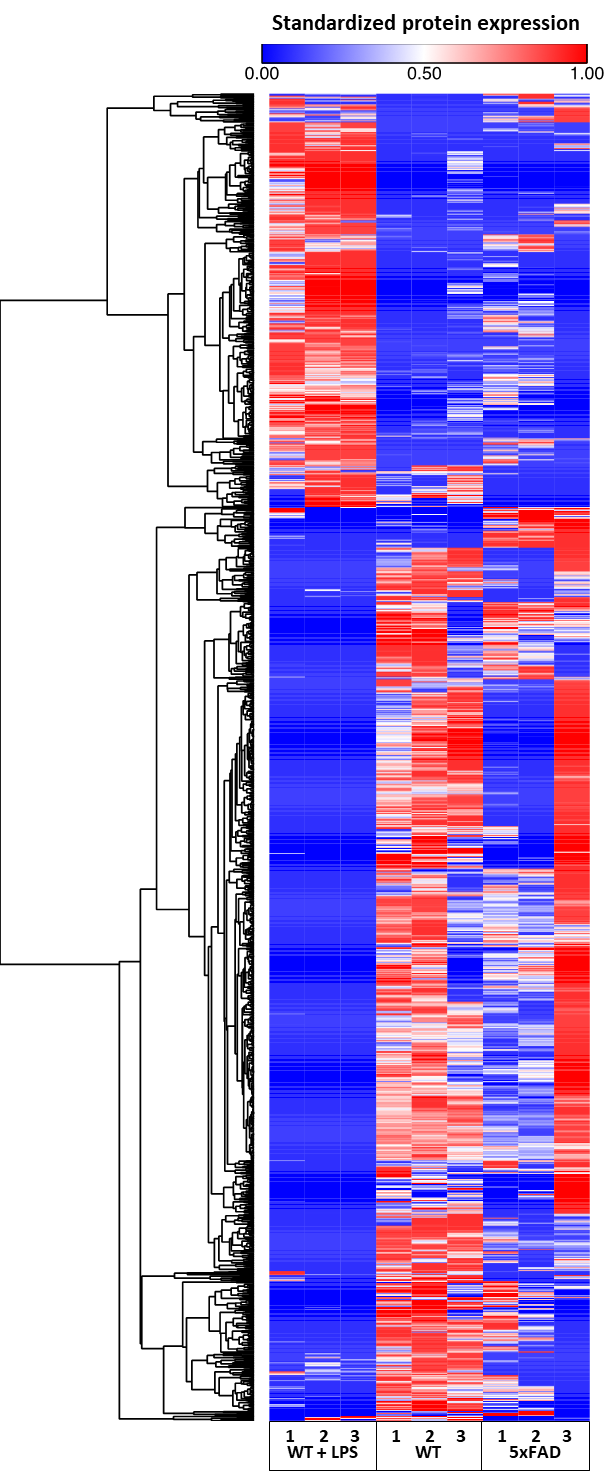


**Figure S1. Hierarchical clustering of proteins differentially expressed (p<0.05) in either WT-LPS vs WT-control and 5xFAD vs WT comparisons.** Absolute protein expression data were standardized ([value-row mean]/standard deviation]) and represented on a color scale of 0-1. Clustering was performed using the one minus Pearson correlation and average linkage method (Morpheus software, Broad institute, <https://software.broadinstitute.org/morpheus/>).

**
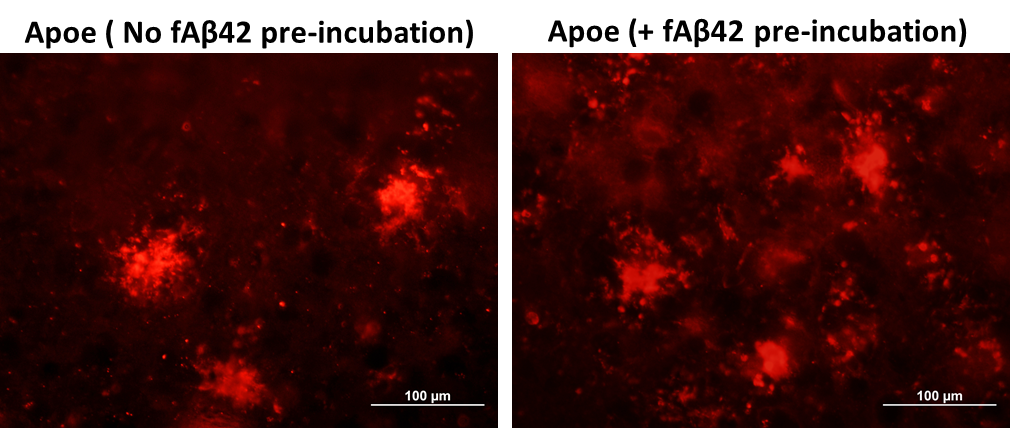
**

**Figure S2. Pre-incubation of anti-Apoe antibody with fibrillar Aβ does not abolish plaque-like immunostaining for Apoe.** Anti-Apoe antibody was first pre-incubated with 200 µM fibrillar Aβ42 for 2 hours after which the sample was centrifuged at 20,000g x 15 min at 4 deg C. 5xFAD brain sections (30µm thickness) were processed as described in the Methods section and then incubated with 1:100 dilution of either pre-incubated or untreated anti-Apoe antibodies (same final effective concentration) overnight, followed by incubation with secondary fluorophore conjugated antibody (1:500 for 1 hour). Immunofluorescence micrographs (40x) from the subiculum regions are shown.
